# A Docking and Network Pharmacology Study on the Molecular Mechanisms of Curcumin in Dental Caries and *Streptococcus mutans*

**DOI:** 10.3390/dj12060153

**Published:** 2024-05-21

**Authors:** Juan Manuel Guzmán-Flores, Ángel Pérez-Reyes, Sonia Isela Vázquez-Jiménez, Mario Alberto Isiordia-Espinoza, Fernando Martínez-Esquivias

**Affiliations:** 1 Departamento de Ciencias de la Salud, División de Ciencias Biomédicas, Centro Universitario de los Altos, Universidad de Guadalajara, Tepatitlán de Morelos 47620, Mexico; 2 División de Ciencias Biomédicas, Centro Universitario de los Altos, Universidad de Guadalajara, Tepatitlán de Morelos 47620, Mexico; angel.perez1047@alumnos.udg.mx (Á.P.-R.); sonia.vazquez@alumnos.udg.mx (S.I.V.-J.); 3 Instituto de Investigación en Ciencias Médicas, Cuerpo Académico Terapéutica y Biología Molecular (UDG-CA-973), Departamento de Clínicas, División de Ciencias Biomédicas, Centro Universitario de los Altos, Universidad de Guadalajara, Tepatitlán de Morelos 47620, Mexico; mario.isiordia@academicos.udg.mx; 4 Departamento de Ciencias Pecuarias y Agrícolas, Centro Universitario de los Altos, Universidad de Guadalajara, Tepatitlán de Morelos 47620, Mexico; fernando.mesquivias@academicos.udg.mx

**Keywords:** pharmacological network, curcumin, caries, *Streptococcus mutans*, bioinformatics

## Abstract

Background: Dental caries is a dynamic, multifactorial disease that destroys teeth and can affect anyone’s quality of life because it can cause tooth loss and make chewing difficult. Dental caries involves various factors, such as *Streptococcus mutans* and host factors. Currently, adjuvant therapies, such as curcumin, have emerged, but how they work has not been adequately described. Therefore, this work aims to identify the molecular mechanism of curcumin in caries and *Streptococcus mutans*. Methods: We obtained differentially expressed genes from a GEO dataset, and curcumin targets were obtained from other databases. The common targets were analyzed according to gene ontology enrichment, key genes were obtained, and binding to curcumin was verified by molecular docking. Results: Our analysis showed that curcumin presents 134 therapeutic targets in caries. According to the gene ontology analysis, these targets are mainly involved in apoptosis and inflammation. There are seven key proteins involved in the action of curcumin on caries: MAPK1, BCL2, KRAS, CXCL8, TGFB1, MMP9, and IL1B, all of which spontaneously bind curcumin. In addition, curcumin affects metabolic pathways related to lipid, purine, and pyrimidine metabolism in *Streptococcus mutans*. Conclusions: Curcumin affects both host carious processes and *Streptococcus mutans*.

## 1. Introduction

Dental caries is a disease caused by a combination of factors such as biofilm, diet, and environmental factors that result in the loss of minerals from the teeth. Due to these interactions, a caries lesion develops; moreover, there are changes in the expression of proteins related to this disease [1,2]. Around the world, dental caries is a significant problem in most industrialized countries, where most children and adults experience this disease [3]. Some methods to prevent dental caries are using fluoridated toothpaste and mouthwash to combat the microbial plaque by chemical methods, diet modification, and preventive interventions by dental professionals. The treatment of dental caries is mainly carried out by a dental surgeon [4].

An alternative form of adjuvant therapy is herbal medicine. It makes use of natural compounds that have both therapeutic and prophylactic properties. These compounds are a safer option compared to antibiotics when it comes to treating and preventing caries due to their specific structures and multiple biological and chemical properties [5]. *Curcuma longa* L. contains phenolic compounds with numerous beneficial activities, including dentin remineralization and antibacterial, anti-inflammatory, and antioxidant properties [5]. Some studies have observed that *Curcuma longa* L. has a dose-dependent antibacterial action and, at high doses, can also inhibit the formation of *Streptococcus mutans* biofilm [6,7].

*Curcuma longa* L. has many active compounds, the most important of which is curcumin. Several studies have shown that curcumin benefits different diseases [8,9,10]. Some studies revealed that curcumin could potentially prevent and be essential in treating dental caries as a natural, accessible, safe, and inexpensive agent to increase oral and dental health [11]. However, the mechanism of action of curcumin in caries has yet to be fully elucidated; moreover, the molecular targets of curcumin have been poorly studied.

Caries dental is a complex disease; most drugs are highly ineffective, and the success rate of drug discovery is in constant decline. Systems, network medicine, and network pharmacology are revolutionizing disease definition, diagnosis, treatment, and cure [12]. Network pharmacology approaches integrate systematic data to analyze the holistic process of interactions between compounds and the human body. Network pharmacology is an emerging discipline that helps to understand the mechanism of action of one or more molecules. This discipline integrates bioinformatics, pharmacological, and molecular biology tools to understand the relationships between drugs, molecular targets, and diseases and to elucidate the mechanism of action of a molecule, group of molecules, or plant compounds [12,13]. Usually, the results obtained from network pharmacology are validated by in vivo, in vitro, or even in silico experiments. One of the most commonly used in silico techniques is molecular docking. Molecular docking is a technique capable of predicting the interactions between a ligand and a target at a molecular level; a degree of affinity is obtained according to the binding energy expressed in ΔG [14].

With a pharmacological network strategy, this work aims to identify the molecular mechanism by which curcumin affects caries and Streptococcus mutans. 

## 2. Materials and Methods

### 2.1. Data Acquisition

We obtained curcumin’s chemical structure and SMILES (simplified molecular input line entry specification) from the PubChem website [15]. Then, we searched for curcumin interactions in various databases, including the Swiss Target Prediction database [16], the Comparative Toxicogenomics Database (CTD) [17], and the STITCH database [18]. We limited our search to the “*homo sapiens*” species to find the targets of curcumin. 

We obtained the target genes for caries from the Gene Expression Omnibus dataset available at the National Center for Biotechnology Information [19]. We found a single dataset related to dental caries, GSE1629 [20]. The research was carried out on two diseased and two healthy samples. The study involved analyzing the expression of core pulpal tissue found in carious teeth. RNA was pooled from 11 carious teeth, and the findings offer a better understanding of the molecular processes that occur during dental caries’ development.

We utilized the GEO2R analysis tool to identify the differentially expressed genes (DEGs). For this analysis, we set the cut-off criteria as *p* < 0.05 and logFC = 0.5. To determine the overlap between curcumin targets and DEGs from the caries datasets, we used the Venny 2.1.0 platform to visualize the results. (https://bioinfogp.cnb.csic.es/tools/venny/ accessed on 2 February 2024).

### 2.2. GO and Pathway Enrichment Analyses

We used the Shiny GO 0.77 tool (http://bioinformatics.sdstate.edu/go/ accessed on 2 February 2024) to identify relevant term enrichment and analyze the shared genes between DEGs and curcumin therapeutic targets. This tool is a graphical web application that allows enrichment analysis of gene ontology and metabolic pathways from a list of genes to be performed. This analysis included Gene Ontology (GO) enrichment for Biological Process (BP), Molecular Function (MF), Cellular Component (CC), and KEEG metabolic pathways [21]. We performed FDR adjustment of *p*-values using the Benjamin-Hochberg method. We limited the results to humans, with a maximum number of pathways of 5000 and a minimum of 2, showing only the top ten.

### 2.3. Construction of the Target-Curcumin-Metabolic Pathways Network in Caries

We used Cytoscape v3.10.1 software [22] to create an interaction network between targets, curcumin, and metabolic pathways. Cytoscape is a popular tool for constructing and visualizing networks, where the nodes represent target proteins, pathways, or molecules, and the edges correspond to predicted or validated interactions between them. Additionally, we determined the key genes based on their connectivity within the network.

### 2.4. Molecular Docking

In our study, we used molecular docking to test the reliability of potential targets for caries treatment, specifically curcumin. We obtained the PBD files of the targets from the AlphaFold Protein Structure Database [23]; we limited the results to the *homo sapiens* organism and took into account only the structures reviewed. The AutoDock Vina program in PyRx 0.9.8 software to perform the simulations [24,25]. We ran eight iterations and selected the best pose for further analysis. The chemical structure of curcumin was obtained from the PubChem database. We visualize the results with Biovia Discovery Studio Visualizer to identify the types of bonds present between proteins and curcumin.

### 2.5. Effect of Curcumin on Streptococcus mutans

We explored the impact of curcumin on *Streptococcus mutans* by analyzing an interaction network between curcumin and bacteria using the STITCH (Search Tool for Interacting Chemicals) database [18]. STITCH is a database that integrates the interactions between proteins and small molecules, such as curcumin, in living organisms; it also provides the results in graphical form and functional enrichment analysis. We tried to determine the possible KEGG pathways affected by curcumin in *Streptococcus mutans*. We set a minimum interaction score of 0.400 for protein-curcumin interaction networks and considered an FDR < 0.05 for the KEGG analysis.

## 3. Results

### 3.1. Analysis of Curcumin Targets and Caries

**Figure 1:**
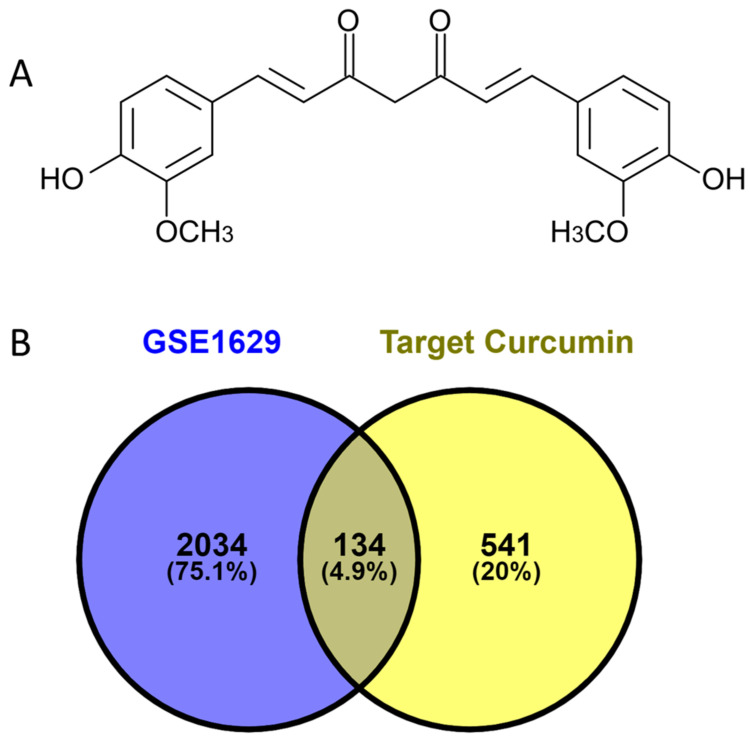
Curcumin and DEGs. (**A**) The chemical structure of curcumin in 2D; (**B**) a Venn diagram of the matched genes between DEGs and curcumin targets.

Figure 1A provides the structure of curcumin. From the analysis of the GSE1629 dataset, we found 2168 DEGs. Concerning curcumin, 675 proteins were identified as therapeutic targets. After matching the DEGs with curcumin targets (Figure 1B), 134 molecules were selected as potential targets for curcumin’s therapeutic effect on dental caries.

### 3.2. Enrichment Analysis of Overlapping Targets

**Figure 2:**
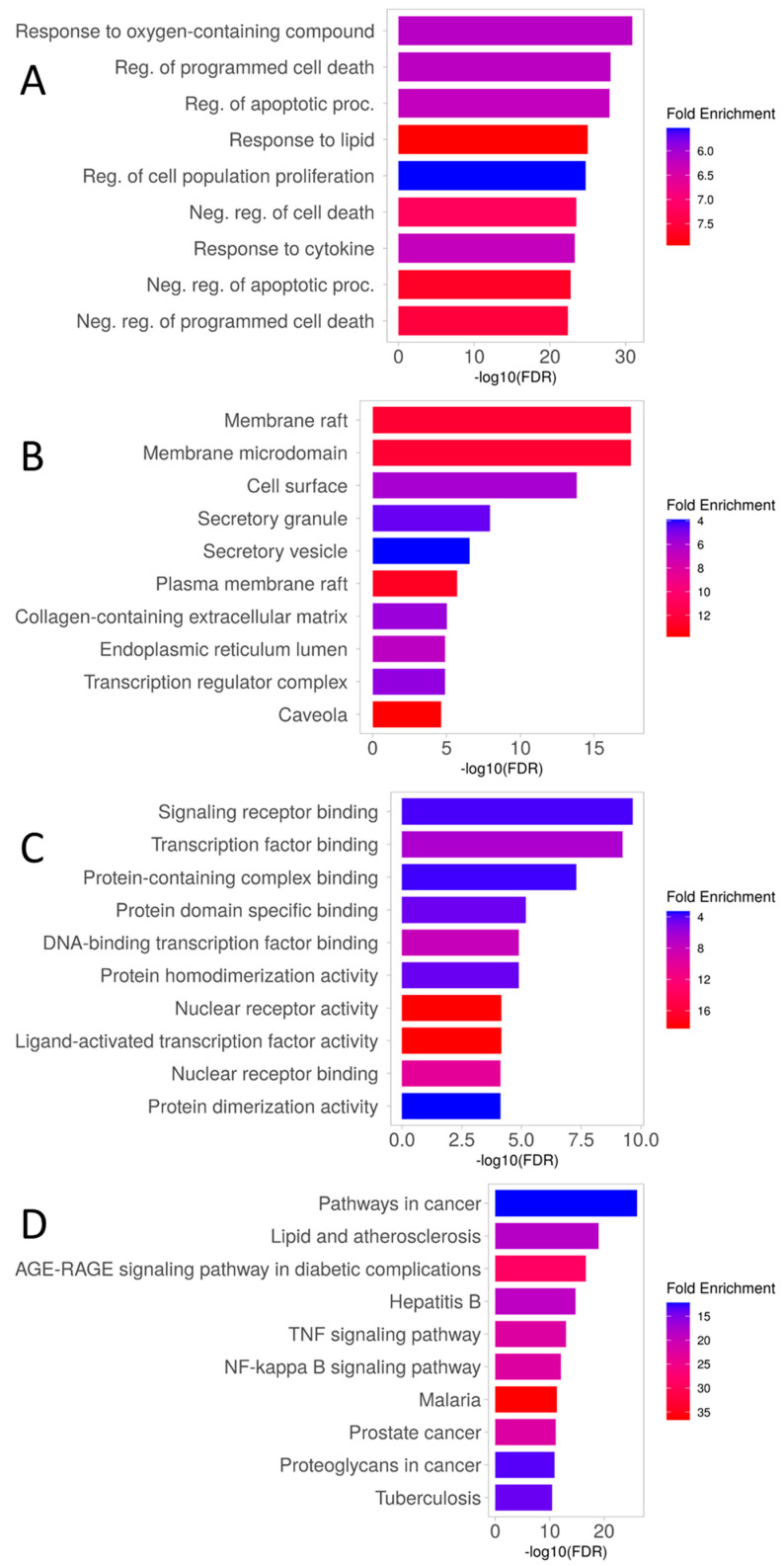
Gene ontology (GO) enrichment analysis for target proteins (top 10). (**A**) Biological process. (**B**) Component Cellular. (**C**) Molecular Function. (**D**) KEEG metabolic pathways.

Gene ontology enrichment analysis for the 134 genes revealed that the most significant biological processes were associated with programmed cell death and cytokine response (Figure 2A). Curcumin’s molecular targets in caries were mainly found in the cell membrane and endoplasmic reticulum (Figure 2B). The enrichment in molecular function was related to transcriptional processes and cell signaling (Figure 2C). Similarly, the enriched metabolic pathways were mainly associated with inflammatory processes, diabetes complications, atherosclerosis, lipids, and various diseases (Figure 2D).

### 3.3. Target-Curcumin-Metabolic Pathways Network in Dental Caries

**Figure 3:**
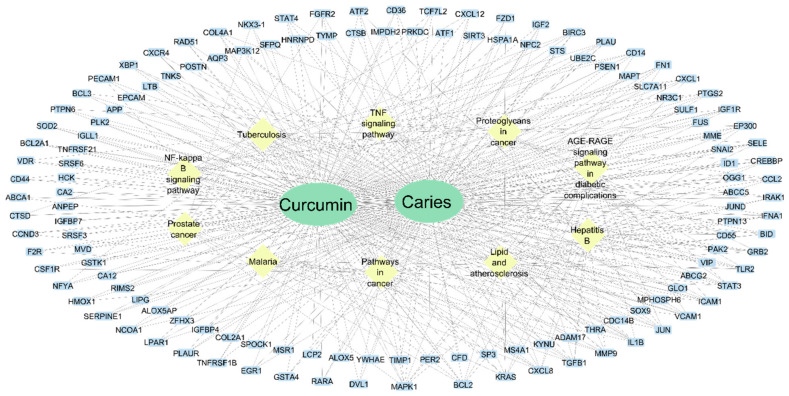
Target-Curcumin-Metabolic pathways network. Yellow diamonds indicate metabolic pathways. The blue rectangles are proteins. The lines indicate interactions.

We constructed a Target-Curcumin-Metabolic pathways network from the 134 common molecules between curcumin targets and genes associated with caries. This network can be seen in Figure 3, which shows that curcumin possibly ameliorates carious lesions by affecting inflammatory metabolic pathways, lipid metabolism, atherosclerosis, and diabetic complications. Furthermore, we can infer that caries is related to other diseases, and curcumin could be a complementary treatment. In this network, we can also observe the proteins with the most interactions: MAPK1, BCL2, KRAS, CXCL8, TGFB1, MMP9, and IL1B, which have 12 to 8 interactions each. Further information on these proteins is shown in Table 1. The information was obtained from the UniProt website.

### 3.4. Validation of Key Proteins Using Molecular Docking

**Figure 4:**
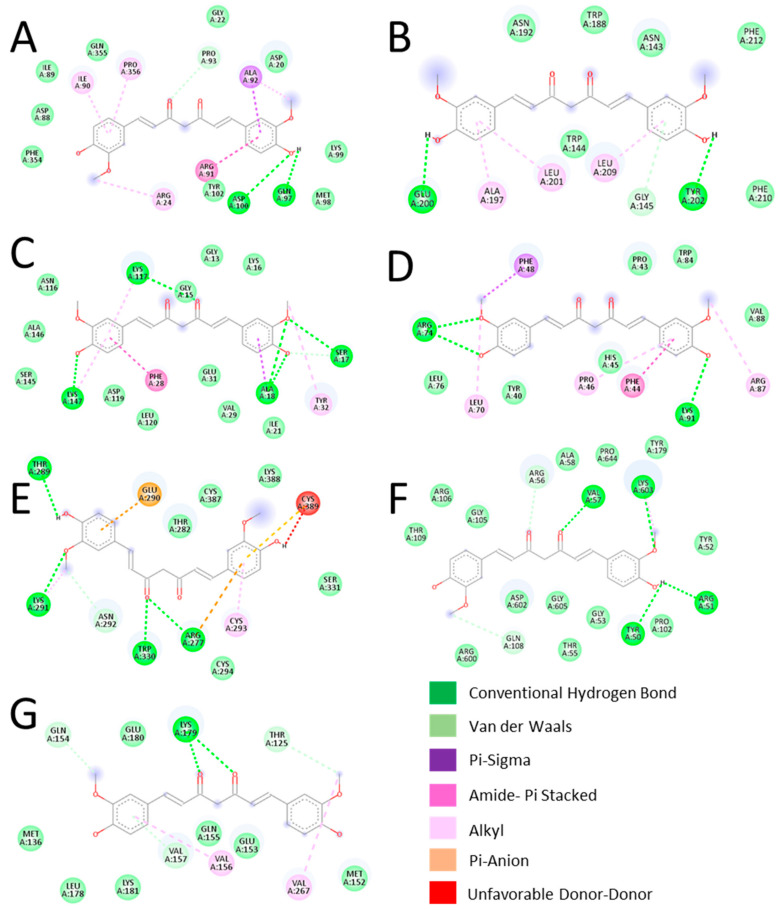
Schematic diagram of the interaction of curcumin and keys proteins with its ΔG expressed in kcal/mol. (**A**) MAPK1, −7.3. (**B**) BCL2, −6.2. (**C**) KRAS, −8.4. (**D**) CXCL8, −6.2. (**E**) TGFB1, −6.7. (**F**) MMP9, −6.8. (**G**) IL1B, −6.7. The different colors indicate the type of bond.

After analyzing the Target-Curcumin-Metabolic Pathways network, we performed a molecular docking simulation to investigate the interaction between curcumin and seven important proteins with the most interactions. Figure 4 displays the simulation outcome; the results showed that the ΔG of all seven proteins was less than 0, suggesting they can bind spontaneously to curcumin. Additionally, Figure 4 depicts the bonds between curcumin and the amino acids of the proteins analyzed. In the seven proteins analyzed, it can be observed that hydrogen bonds exist between the amino acids of the proteins and curcumin. Interestingly, KRAS had the lowest ΔG of all proteins.

### 3.5. Effect of Curcumin on Streptococcus mutans

**Figure 5:**
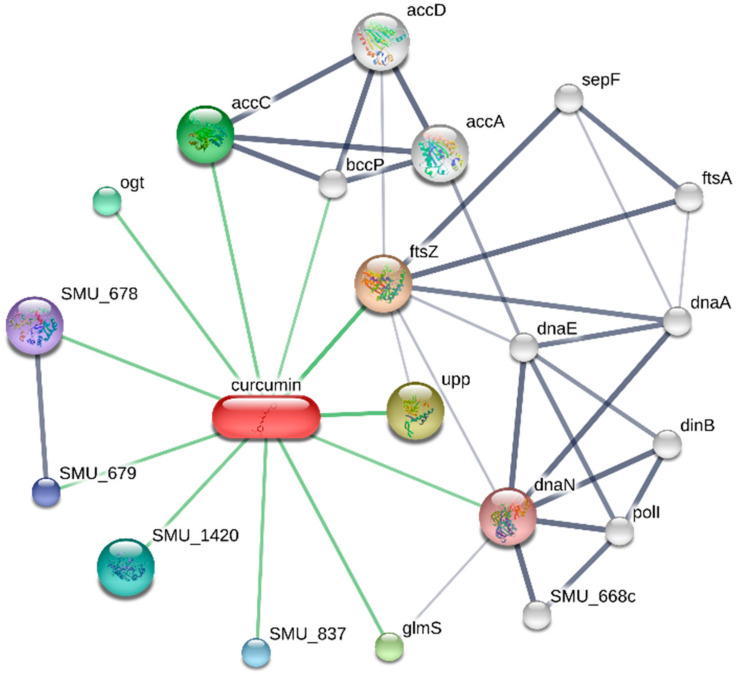
Interaction network between curcumin and *Streptococcus mutans* proteins.

One etiological factor in caries is infection by *Streptococcus mutans* bacteria. Therefore, we decided to investigate the effect of curcumin on these bacteria through an *in silico* approach. The interaction network between curcumin and *Streptococcus mutans* self-proteins is shown in Figure 5. Although few interactions were observed in this network, enrichment analysis of the KEGG Pathways yielded 11 enriched metabolic pathways, which are Fatty acid biosynthesis, Propanoate metabolism, Fatty acid metabolism, Pyrimidine metabolism, Pyruvate metabolism, DNA replication, Homologous recombination, Purine metabolism, Metabolic pathways, Carbon metabolism, and Biosynthesis of secondary metabolites. Therefore, it can be stated that curcumin exerts its effect on *Streptococcus mutans* through these metabolic pathways.

## 4. Discussion

Dental caries is a current disease that impacts all age groups, causing pain, suffering, impairment of function, and reduced quality of life. Its treatment costs are not always well covered by most of the population, resulting in the loss of teeth [1,3]. In this work, we investigate the molecular mechanisms of curcumin in dental caries through a network pharmacology approach. Our results confirm that curcumin regulates metabolic pathways related to the immune system and signaling cytokines. We found seven genes through which curcumin could exert several biological activities. We establish that the product of these genes, proteins, is highly likely to bind to curcumin spontaneously. In addition, we identified some metabolic pathways through which curcumin exerts its action on *Streptococcus mutans*.

Gene ontology enrichment showed that the biological process of apoptosis is related to curcumin in dental caries. Previous reports have shown that apoptosis is active in caries; furthermore, although fluoride can prevent caries, excessive fluoride intake can cause fluorosis. However, curcumin attenuates fluoride-mediated apoptosis through Akt activation and suppresses fluoride-mediated DNA damage [26,27]. Therefore, curcumin may reverse the apoptotic process caused by dental caries and excessive fluoride intake. Another biological process resulting from gene ontology enrichment was the response to lipids. It has been reported that one of the main virulence factors of *Streptococcus mutans* is the specific composition of lipids present in its membrane, which changes depending on the pH [28], so it is interesting that curcumin regulates the molecules involved in the lipid response. 

It is also interesting to mention that curcumin acts on the cell surface, specifically on the cell membrane, as well as on granules or secretory vesicles. On the other hand, molecular functions related to curcumin action and caries involve binding to signaling receptors and transcription factors, events possibly associated with the TNF signaling pathway, AGE-RAGE, and NF-κB activation.

Enrichment of metabolic pathways showed that curcumin is related to immune processes in caries, including the TNF signaling pathway and the AGE-RAGE axis. This cytokine has been reported to be increased in subjects with caries, and a polymorphism in this gene has also been associated with caries [29,30]. Similarly, RAGE mRNA levels have increased in the inflamed pulp of carious teeth [31]. This demonstrates the inflammatory process present in caries. On the other hand, it is well known that curcumin possesses potent anti-inflammatory effects, including decreasing circulating levels of TNF and decreasing NF-κB expression [32,33]. Due to the above, it is reasonable to assume that curcumin is anti-inflammatory in carious lesions.

According to their degree, the key proteins involved in the metabolic pathways related to curcumin’s action on caries were MAPK1, BCL2, KRAS, CXCL8, TGFB1, MMP9, and IL1B. The interaction between these proteins and curcumin was verified by molecular docking analysis, resulting in a favorable interaction between all proteins, especially KRAS. 

MAPK proteins play an important role in proliferation, differentiation, development, transformation, and apoptosis, and they exert their action when phosphorylated. MAPK1 protein has been reported to be overexpressed in cultured human dental pulp cells from decayed teeth [34]. Curcumin has been shown to inhibit MAPK1 phosphorylation in cultured pancreatic beta cells [35]. Therefore, curcumin could decrease caries inflammation through MAPK1. BCL2 is a protein that regulates cell death and has been reported to be overexpressed in teeth with advanced caries [26]. However, it has also been reported that curcumin ameliorates bone loss by inhibiting osteoclastogenesis through several molecules, including BCL2 [36].

Another key protein in curcumin’s effect on caries was KRAS, which regulates cell proliferation. KRAS was the protein with the lowest ΔG, possibly due to the higher number of hydrogen bonds formed between the two molecules. To our knowledge, this protein has not been related to caries but has been reported to be associated with odontogenic lesions. Notably, curcumin can regulate this protein [37,38].

MMP9 is a protein of the matrix metalloproteinase family that plays an essential role in the proteolysis of the extracellular matrix. A study of the salivary proteome of children with caries identified this differentially expressed protein [39]. Similarly, another study showed that curcumin can suppress MMP9 [40].

CXCL8, also called IL-8, is a chemotactic factor involved in the inflammatory response by attracting neutrophils, basophils, and T cells to eliminate pathogens and protect the host from infection, whereas IL1B is a potent proinflammatory cytokine. These two immune molecules have been associated with caries. CXCL8 has been reported to be overexpressed in carious lesions, and a genetic variant of IL1B has been associated with caries susceptibility, and both have been reported as therapeutic targets of curcumin in periodontitis [9,41,42]. Therefore, according to previous reports, the seven key proteins can be regulated by curcumin.

It is well known that *Streptococcus mutans* is involved in developing dental caries. At the beginning of the infection, these bacteria metabolize sugars, causing acidification in the medium. It has been shown that *Streptococcus mutans* significantly changes the proportion of fatty acids in its membrane in response to environmental acidification, which involves fatty acid metabolism [43]. Our analyses show that curcumin can affect fatty acid metabolism. Therefore, curcumin’s antibacterial effect may involve this metabolic pathway [7].

Other metabolic pathways by which curcumin affects *Streptococcus mutans* are carbon and pyruvate metabolism. These two pathways are essential for the bacteria’s survival because they are its primary energy sources [44]. The last metabolic pathways affected by curcumin in *Streptococcus mutans* are DNA replication and purine and pyrimidine metabolism. It is reasonable to assume that any alteration in these metabolic pathways will result in the death of the bacteria and, consequently, curcumin’s antibacterial effect.

Finally, we know that this work’s results should be verified experimentally. However, it is also important to mention that the effect of curcumin on caries and *Streptococcus mutans* has been extensively studied, and we are contributing to this field by proposing the mechanism by which curcumin carries out the beneficial effects on the carious process and the bacteria. Another limitation of this study is that, with this experimental design, it is impossible to calculate the curcumin dose to achieve its therapeutic effect. It is also important to mention that the pharmacological effect of curcumin can be influenced by other nutraceuticals consumed simultaneously.

## 5. Conclusions

Based on our findings, we can conclude that curcumin exerts its therapeutic effect on caries through 134 targets, which are involved in apoptosis and inflammatory processes. There are seven key proteins involved in the action of curcumin on dental caries: MAPK1, BCL2, KRAS, CXCL8, TGFB1, MMP9, and IL1B, all of which spontaneously bind curcumin. In addition, curcumin also affects the metabolic pathways of *Streptococcus mutans*, such as fatty acid, pyruvate, purine, and pyrimidine metabolism, as well as DNA replication. Therefore, curcumin affects both host carious processes and *Streptococcus mutans*.

## Figures and Tables

**Table 1:** Proteins with the highest number of interactions.

Gene	Protein	Function
MAPK1	Mitogen-activated protein kinase 1	MAPK1 is involved in cell growth, adhesion, survival, and differentiation through the regulation of transcription, translation, and cytoskeletal rearrangements.
BCL2	Apoptosis regulator Bcl-2	Regulates cell death by controlling the mitochondrial membrane permeability.
KRAS	KRAS proto-oncogene, GTPase	It plays an important role in the regulation of cell proliferation
CXCL8	Interleukin-8	Chemotactic factor that mediates inflammatory response by attracting neutrophils, basophils, and T-cells to clear pathogens and protect the host from infection.
TGFB1	Transforming growth factor beta-1	Plays an important role in bone remodeling: acts as a potent stimulator of osteoblastic bone formation, causing chemotaxis, proliferation, and differentiation in committed osteoblasts.
MMP9	Matrix metalloproteinase-9	Could play a role in bone osteoclastic resorption.
IL1B	Interleukin-1 beta	It induces prostaglandin synthesis, neutrophil influx and activation, T-cell activation and cytokine production, B-cell activation and antibody production, and fibroblast proliferation and collagen production.

## Data Availability

The original contributions presented in the study are included in the article, further inquiries can be directed to the corresponding author.
